# Mitochondrial Translocator Protein (TSPO) Expression in the Brain After Whole Body Gamma Irradiation

**DOI:** 10.3389/fcell.2021.715444

**Published:** 2021-10-25

**Authors:** Calina Betlazar, Ryan J. Middleton, Nicholas Howell, Ben Storer, Emma Davis, Justin Davies, Richard Banati, Guo-Jun Liu

**Affiliations:** ^1^Australian Nuclear Science and Technology Organisation, Sydney, NSW, Australia; ^2^Discipline of Medical Imaging and Radiation Sciences, Faculty of Medicine and Health, Brain and Mind Centre, University of Sydney, Camperdown, NSW, Australia

**Keywords:** translocator protein, neuroinflammation, radiobiology, microglia, endothelial cells

## Abstract

The brain’s early response to low dose ionizing radiation, as may be encountered during diagnostic procedures and space exploration, is not yet fully characterized. In the brain parenchyma, the mitochondrial translocator protein (TSPO) is constitutively expressed at low levels by endothelial cells, and can therefore be used to assess the integrity of the brain’s vasculature. At the same time, the inducible expression of TSPO in activated microglia, the brain’s intrinsic immune cells, is a regularly observed early indicator of subtle or incipient brain pathology. Here, we explored the use of TSPO as a biomarker of brain tissue injury following whole body irradiation. Post-radiation responses were measured in C57BL/6 wild type (*Tspo*^+/+^) and TSPO knockout (*Tspo*^–/–^) mice 48 h after single whole body gamma irradiations with low doses 0, 0.01, and 0.1 Gy and a high dose of 2 Gy. Additionally, post-radiation responses of primary microglial cell cultures were measured at 1, 4, 24, and 48 h at an irradiation dose range of 0 Gy-2 Gy. TSPO mRNA and protein expression in the brain showed a decreased trend after 0.01 Gy relative to sham-irradiated controls, but remained unchanged after higher doses. Immunohistochemistry confirmed subtle decreases in TSPO expression after 0.01 Gy in vascular endothelial cells of the hippocampal region and in ependymal cells, with no detectable changes following higher doses. Cytokine concentrations in plasma after whole body irradiation showed differential changes in IL-6 and IL-10 with some variations between *Tspo^–/–^* and *Tspo*^+/+^ animals. The *in vitro* measurements of TSPO in primary microglial cell cultures showed a significant reduction 1 h after low dose irradiation (0.01 Gy). In summary, acute low and high doses of gamma irradiation up to 2 Gy reduced TSPO expression in the brain’s vascular compartment without *de novo* induction of TSPO expression in parenchymal microglia, while TSPO expression in directly irradiated, isolated, and thus highly activated microglia, too, was reduced after low dose irradiation. The potential link between TSPO, its role in mitochondrial energy metabolism and the selective radiation sensitivity, notably of cells with constitutive TSPO expression such as vascular endothelial cells, merits further exploration.

## Introduction

Ionizing radiation is widely used for both diagnostic and therapeutic purposes in medicine. Despite the prevalence of its application, there remain gaps in our knowledge, notably, of the neurobiological responses to diagnostically relevant low dose ionizing radiation (LDIR) (Betlazar et al., 2016; Tang et al., 2017). The issue is also of interest as there is evidence pointing to non-linear differences in the responses to both low and high dose ionizing radiation (Tang and Loke, 2015).

Microglial cells, the intrinsic tissue macrophages of the brain, are key to the understanding of the brain’s injury responses, including those caused by ionizing radiation (Acharya et al., 2016; Hladik and Tapio, 2016; Krukowski et al., 2018). Higher doses of ionizing radiation at 2 Gy and above cause microglial activation (Hwang et al., 2006; Voloboueva et al., 2010; Chen et al., 2016), associated with cognitive changes and synaptic loss over the longer term (Krukowski et al., 2018; Allen et al., 2020). Some of the earliest and most important responses to ionizing radiation are the generation of increased reactive oxygen species (ROS) and oxidative stress (Kam and Banati, 2013; Szumiel, 2015; Richardson and Harper, 2016; Farhood et al., 2019) produced by mitochondria, including in macrophages, as part of radiation-induced inflammatory tissue responses (Banati et al., 2004; Simpson and Oliver, 2020). For example, doses of 0.5 Gy have been shown to increase ROS in microglial cell lines (Chen et al., 2016), and x-ray doses of 2 Gy induced microglial activation in the hippocampus, modulating the activity of electron transport chain enzymes in mitochondria (Casciati et al., 2016). Even higher doses have been shown to induce oxidative damage, as well as the expression of mitochondrial fission and fusion proteins concomitantly with microglial activation (Zhou et al., 2017).

However, there is still a paucity of studies examining neurobiological responses to LDIR, specifically, microglial and inflammatory responses. A recent study investigated very low dose total body exposure of 0.063–0.5 Gy gamma radiation in mice and found that the lowest dose of 0.063 Gy was able to reduce the number of activated microglia (Iba1 expression) in the hippocampus compared to controls 24 months after irradiation, which the authors concluded was indicative of an anti-inflammatory effect. Even low doses of 0.125 and 0.5 Gy were able to induce higher oxidative stress, measured through carbonylated proteins, and 0.5 Gy also upregulated Nrf2 expression, thereby modulating antioxidant responses (Hladik et al., 2019). Evidently, LDIR is able to modulate inflammatory pathways and microglial activity. However, the reported data relate to the long-term consequences of LDIR exposure rather than the immediate effects of LDIR which are underexplored, despite the fact that acute LDIR exposure is common, particularly in a medical diagnostic setting.

The mitochondrial translocator protein (18 kDa; TSPO) is an outer mitochondrial membrane protein that is expressed in activated, but not resting, microglia during nerve injury or other active brain pathologies, and has therefore been used as an indicator of microglial activation (Liu et al., 2014; Gatliff and Campanella, 2016; Cumming et al., 2018; Betlazar et al., 2020; Ullah et al., 2020). Recent evidence suggests that TSPO may play a fundamental role in the innate immune response, underpinned by its involvement in mitochondrial energy metabolism (Banati et al., 2014; Liu et al., 2017; Betlazar et al., 2020), as well as ROS formation and modulation in response to various stress stimuli (Choi et al., 2011; Gatliff et al., 2014; Gatliff and Campanella, 2015; Pozzo et al., 2019; Betlazar et al., 2020; Fu et al., 2020). TSPO interacts with the key drivers of inflammation and ROS activation, such as the NOX enzymes (Guilarte et al., 2016; Loth et al., 2020), as well as downstream signaling pathways and transcriptional regulators such as NF-kB, AP-1, and the MAPK pathway (Batarseh et al., 2010; Zhao et al., 2011; Horiguchi et al., 2019; Pozzo et al., 2019). TSPO is generally expressed in mitotic cells other than activated microglia, including vascular endothelial cells, ependymal cells and neural stem cells, consistent with its putative function in mitochondrial energetics (Betlazar et al., 2018; Notter et al., 2018b, 2020). Apart from diagnostic applicability, TSPO can also be targeted for therapeutic purposes for brain injury and brain diseases (Dimitrova-Shumkovska et al., 2020; Veenman, 2020). Here, we explore the utility of TSPO expression as a cellular measure of the impact of low and high doses of ionizing radiation on the brain.

## Materials and Methods

### Animals and Irradiation Procedure

Six-week old male wild type C57BL/6 (*Tspo*^+/+^) mice were obtained from the Animal Resource Centre (Western Australia). TSPO knockout mice (*Tspo*^–/–^) with C57BL/6 genetic background [C57BL/6-*Tspo*^*Tspotm*1G*uWu*^ ^(GuwiyangWurra)^] were bred at the Australian Nuclear Science and Technology Organization (ANSTO) as previously described (Banati et al., 2014). All animal procedures were approved by the University of Sydney Animal Ethics Committee and the ANSTO Animal Care and Ethics Committee. All procedures were in accordance with the Australian Code of Practice for the Care and Use of Animals for Scientific Purposes (8th edition, 2013), and comply with the ARRIVE guidelines. At 6–8 weeks, mice were placed in individual compartments within a Perspex holder and exposed to a single whole-body dose of gamma irradiation under a Cobalt-60 source (ANSTO). A dose rate of 0.0718 Gy/min was used to deliver a final absorbed dose of 0.01 Gy, 0.1 Gy, or 2 Gy (*n* = 10/group). Sham-irradiated (0 Gy) controls were placed in the Perspex holder only. Forty-eight hours after irradiation, mice were euthanized by isofluorane overdose and exsanguinated. Blood was collected by cardiac puncture into EDTA blood collection tubes and centrifuged at 1,500 g for 10 min at room temperature. Blood plasma was collected into microtubes and stored at −80°C until use. Brains were removed and snap frozen in liquid nitrogen and kept at −80°C until sectioning.

Brain tissue coronal sections were cut at 10 μm thickness on a cryostat (Leica CM3050) at −20°C according to the Paxinos and Franklin Mouse brain atlas (Paxinos and Franklin, 2004). Coronal tissue sections were collected from the expanse of the hippocampal region. Sections were mounted onto Poly-L-lysine slides (Thermo Fisher Scientific, Waltham, MA, United States) and slides were stored at −80°C until use.

### Culture and Irradiation of Primary Microglial Cells

Primary microglia were cultured from 0 to 2 day old C57BL/6 mice as previously described (Banati et al., 2014). Briefly, whole brains were dissected and finely cut, then treated with 0.025% trypsin (Sigma-Aldrich, St. Louis, MO, United States). Cells were cultured in Dulbecco’s modified eagle’s medium (DMEM/F12; Sigma Aldrich) supplemented with 10% fetal bovine serum (Thermo Fisher Scientific), 1% penicillin-streptomycin-glutamine (Sigma-Aldrich) and 0.5 ng/mL GM-CSF (Abcam, Cambridge, United Kingdom). Microglia in the medium were collected after shaking at 350 r.p.m. for 1 h at 37°C. After pelleting the cells by centrifugation, cells were suspended in the DMEM/F12 media but without GM-CSF. After 15 min the cells were washed twice with the supplemented DMEM to remove unattached cells. The > 98% purity of microglial cultures was confirmed by staining with the microglial marker Alexa Fluor 488-conjugated isolectin GS-IB4 (Thermo Fisher Scientific).

Primary cells were seeded at 5 × 10^4^ density onto poly-L-lysine coated 13 mm round coverslips in 24 well plates and grown for 2 days before gamma irradiation. Cells underwent single dose exposure to gamma radiation at 0.01, 0.1, and 2 Gy. Sham-irradiated cells (0 Gy) were not exposed to radiation. At pre-determined time points post-irradiation (1, 4, 24, and 48 h), cells were fixed with 3.7% paraformaldehyde (Sigma-Aldrich) for 10 min, then coverslips were kept at 4°C in PBS until subsequent immunocytochemistry.

### Immunocytochemistry

Primary microglial cells were fixed with 3.7% formaldehyde in PBS for 10 min, permeabilized with 0.5% Triton X-100 solution (Sigma-Aldrich), then incubated with an anti-PBR/TSPO antibody (Abcam, #ab109497) in 5% bovine serum albumin (BSA) for 45 min at 37°C. Coverslips were washed with PBS, then incubated with Alexa Fluor 594-conjugated goat anti-rabbit IgG (Abcam) for 30 min at 37°C. Coverslips were mounted on slides with ProLong Diamond Antifade Mountant with DAPI (Thermo Fisher Scientific) and images were captured with a BX61W1 Olympus microscope. The images were then deconvoluted with the AutoQuant X3 program (Media Cybernetics, Rockville, MD, United States).

### Immunohistochemistry/Immunofluorescence

Immunohistochemistry was performed as previously described (Banati et al., 2014; Betlazar et al., 2018). Tissue sections were fixed with 3.7% formaldehyde in PBS for 5 min, then permeabilized with ice-cold acetone. Non-specific antibody binding was blocked with 5% goat serum and 5% BSA in PBS. Sections were incubated with an anti-PBR/TSPO antibody (Abcam, #ab109497) at 4°C overnight. After three washes with PBS (10 min/wash), sections were then incubated with a secondary biotinylated goat anti-rabbit antibody at room temperature for 1 h using the Vectastain ABC kit (Vector Laboratories, Burlingame, CA, United States). After three washes, sections were then incubated with the ABC solution, and developed using a metal-enhanced 3, 3′-diaminobenzidine tetrahydrochloride DAB substrate kit (Thermo Fisher Scientific). Sections were then dehydrated in a graded series of ethanol followed by xylene (Sigma-Aldrich), and were then mounted and coverslipped in DPX mounting medium (Sigma-Aldrich).

Immunofluorescence staining was performed as previously described (Betlazar et al., 2018). Tissue sections were fixed with 3.7% formaldehyde in PBS for 5 min, then permeabilized with ice-cold acetone. Non-specific antibody binding was blocked with 5% goat serum and 5% BSA in PBS. Sections were incubated with anti-PBR/TSPO (Abcam, #ab109497) at 4°C overnight. After three washes with PBS (10 min/wash), sections were then incubated with Alexa Fluor 594-conjugated goat anti-rabbit IgG (Abcam) for 1 h at room temperature. ProLong Diamond Antifade Medium with DAPI (Thermo Fisher Scientific) was applied to slides, then coverslipped after three washes. Whole brain sections were imaged using an Olympus VS120 Virtual Slide Microscope (Olympus, Tokyo, Japan). Images were then processed with ImageJ software (NIH, Baltimore, MD, United States).

### Fluorescence and Area Percentage Quantification

Brain tissue sections from the hippocampal level of *Tspo*^+/+^ animals were used to measure the area percentage of positive TSPO expression using DAB staining and TSPO staining immunofluorescence intensity. Sections were analyzed from the same staining batches, same region and same level of the hippocampus. All images were consistently thresholded using ImageJ to find area% positive TSPO expression in pre-defined regions of interest in the hippocampus. Fluorescence intensity of TSPO staining was measured after background subtraction for regions of interest using ImageJ.

### ELISA

Cytokines IL-6 and IL-10 were chosen to reveal any pro- or anti-inflammatory effects of irradiation. Cytokine expression in blood plasma samples from *Tspo*^+/+^ and *Tspo*^–/–^ mice were measured using an IL-6 ELISA kit (Abcam) and IL-10 ELISA kit (R&D, Minneapolis, Minnesota, United States) according to the manufacturer’s instructions. A 50 μL volume of plasma for 6–8 samples per group (1:2 dilution) and standards of known IL-6 and IL-10 concentrations were added into each well of a 96 well plate. The intensity of the absorbance, measured with a SpectraMax^®^ i3x multi-mode microplate reader (Molecular Devices, San Jose, CA, United States), is directly proportional to the concentration of IL-6 and IL-10 present in the plasma.

### RNA Extraction and RT-qPCR

To isolate total RNA, brain tissue was homogenized in TRIzol (Thermo Fisher Scientific) with the lysates then processed using the PureLink RNA Mini Kit (Thermo Fisher Scientific). To remove residual DNA, an on-column DNase treatment was performed (Thermo Fisher Scientific). RNA concentration was measured using a NanoDrop 2000c Spectrophotometer (Thermo Fisher Scientific) and RNA integrity for each sample was assessed by agarose gel electrophoresis.

Purified RNA (1 μg) was reverse transcribed using oligo(dT) primers from the SuperScript III First-Strand Synthesis kit (Thermo Fisher Scientific). cDNA was further diluted with nuclease-free water at a 1:20 dilution for RT-qPCR. The diluted cDNA was added to 2.5 μL of SsoFast EvaGreen Supermix (Bio-Rad, Hercules, CA, United States) and 0.5 μM of each primer pair (Supplementary Table 1) in a final reaction volume of 5 μL. The PCR conditions for both targets were 98°C for 30 s, followed by 45 cycles at 98°C for 5 s, and 63°C for 10 s. Primer specificity was confirmed by analysis of PCR products using agarose gel electrophoresis and sequencing (AGRF, Sydney, Australia). *Gapdh* and β*-actin* were used as reference genes for normalization, and all samples were run in triplicate using a CFX 384 Real-Time PCR Detection System (Bio-Rad, Hercules, CA, United States). A melt curve analysis was performed to confirm the specificity of each of the reactions. Cq values were calculated using the CFX Manager Software (Bio-Rad) with relative expression determined using the Pfaffl method (Pfaffl, 2001).

### Western Blotting

Single-hemisphere brain samples were homogenized in a solution of 188 mM NaCl, 62.5 mM Tris-HCl (pH 7.6) and complete EDTA-free protease inhibitors (Roche) using a bead beater at 5 m/s for 20 s. Triton X-100, sodium deoxycholate (Sigma-Aldrich) and SDS (Sigma-Aldrich) were added to the homogenized samples to make RIPA lysis buffer with a final concentration of 150 mM NaCl, 1.0% Triton X-100, 0.5% sodium deoxycholate, 0.1% SDS, and 50 mM Tris-HCl. Additional RIPA lysis buffer was added and the samples incubated with constant agitation for 2 h at 4°C. Protein concentration was determined using a microplate protein BCA assay (Thermo Fisher Scientific).

Laemmli 4x sample buffer (Bio-Rad) with 200 mM DTT was added to 30 μg of protein before being heated at 70°C for 10 min to denature the proteins. Samples were loaded onto SDS-polyacrylamide gels (Bio-Rad) with 5 μL Precision Plus Protein Western Blotting Standard (Bio-Rad). The separated proteins were transferred onto Bio-Rad 0.2 μm nitrocellulose membranes (Bio-Rad) using the Bio-Rad TransBlot Turbo’s mini-TGX pre-programmed protocol. Immediately after transfer, membranes were immersed in Ponceau S staining solution (Sigma-Aldrich) for 2 min before being rinsed in ddH_2_O and imaged for 0.167 s using the ImageQuant LAS 4000 Digital Imager.

Membranes underwent primary incubation with the relevant primary antibody: TSPO (Abcam), Iba1 (Abcam) and GAPDH (Abcam). Primary antibodies were diluted at 1:1,000 in 5% non-fat dried milk powder in tris-buffered saline with 0.1% Tween 20 Detergent (TTBS) and incubated overnight at 4°C. The secondary antibody (anti-rabbit IgG; Sigma-Aldrich) was diluted 1:1,000 in 5% non-fat dried milk powder in TTBS, along with 1:10,000 Streptactin-HRP conjugate (Bio-Rad), and incubated on membranes for 1 h at room temperature with gentle agitation. Membranes were incubated for 5 min in SuperSignal West Pico PLUS Chemiluminescent Substrate (Thermo Fisher Scientific) with blot images collected using the chemiluminescent function of the ImageQuant LAS 4000 Digital Imager under precision exposure for 15 min.

Densitometric analysis of both total lane density and target protein density was performed using ImageJ. GAPDH data was used to compare the total protein normalization. Total lane density, used as the loading control, was determined using the signal intensity of all proteins in a lane, using the Ponceau S stained images. Target protein densities were determined from the signal intensities of target proteins in each lane, using chemiluminescent images. Fold difference was calculated to determine the quantity of target proteins in each sample normalized to total lane density and density of the protein in the control sample used across each membrane, according to a previously published method (Taylor and Posch, 2014).

### Statistical Analyses

Statistical analyses were performed using GraphPad Prism 7.0b (La Jolla, CA, United States). Statistically significant differences in TSPO and Iba1 expression between radiation groups were determined through one-way analysis of variance (ANOVA) with Dunnett’s *post hoc* test. Genotype and radiation effects were analyzed with a two-way ANOVA followed by Tukey’s *post hoc* test. A *p*-value < 0.05 was considered statistically significant.

## Results

### LDIR Downregulates Brain TSPO and mRNA Expression

**FIGURE 1:**
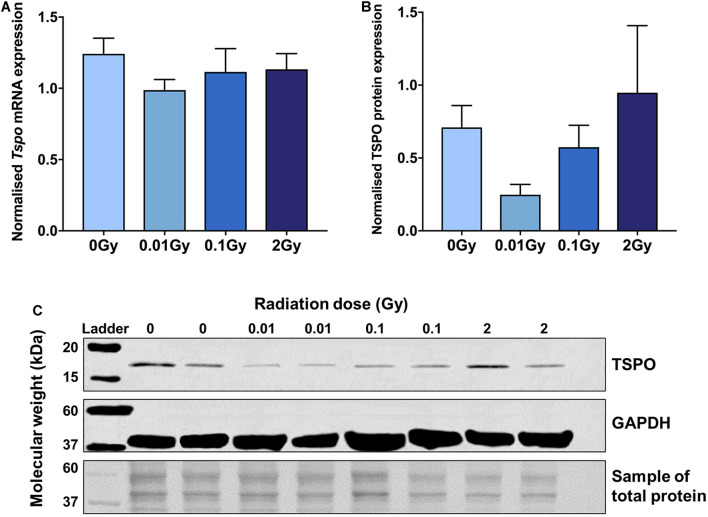
TSPO protein and mRNA expression in brain tissue following whole body gamma irradiation demonstrates a downregulation after 0.01 Gy exposure. **(A)** *Tspo* mRNA expression (normalized to *Gapdh* and *Actin*) decreased after 0.01 Gy whole body irradiation compared to control, though did not reach statistical significance (*p* > 0.05). **(B,C)** Western blotting (normalized to total protein concentration) confirmed a downregulation in TSPO protein expression after 0.01 Gy irradiation, but also did not reach significance (*p* > 0.05). Blot images have been merged using bands from different membranes. Whilst the entire membrane was used for total protein normalization, only a section of the membrane is displayed in this image. Data are presented as mean and standard error, *n* = 3–5/group.

RT-qPCR and western blotting were performed on whole brain tissue collected 48 h after irradiation, to capture peak microglial activity after acute irradiation (Figure 1). A downregulation of TSPO expression was found after 0.01 Gy for both mRNA and protein compared with controls, though this did not reach significance. Decreases in TSPO mRNA and protein expression levels compared to controls were also seen after 0.1 Gy irradiation, along with an increase in TSPO protein expression after 2 Gy, though these also did not reach statistical significance (*p* > 0.05). A smaller trend toward reduced expression was found for mRNA and protein expression of Iba1 (Supplementary Figure 1). Overall, these results suggest a subtle downregulation in TSPO expression and microglial reactivity after LDIR exposure.

### LDIR Downregulates Microglial TSPO Expression *in vitro*

**FIGURE 2:**
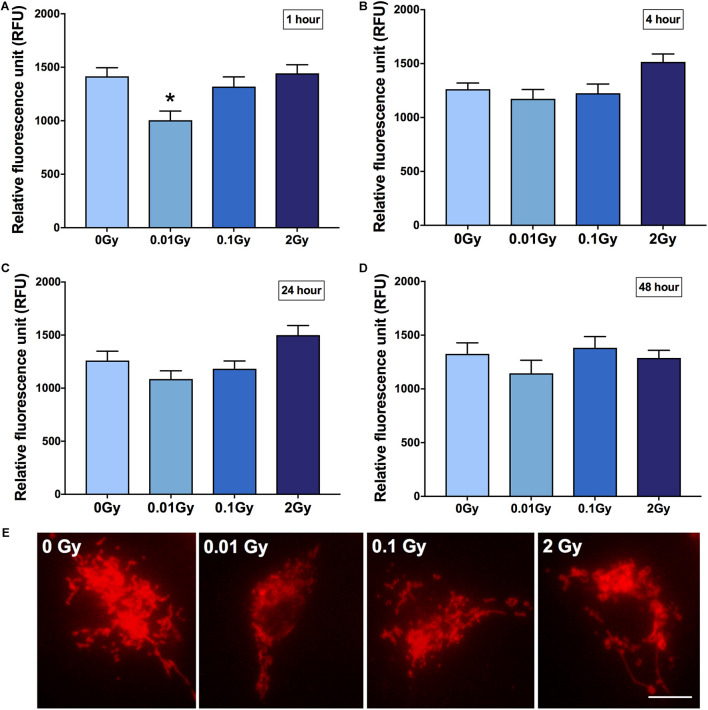
TSPO expression in primary cultured microglia after gamma radiation exposure. Primary microglial cell cultures were exposed to 0.01, 0.1, or 2 Gy plus a sham-irradiated control (0 Gy). TSPO immunoreactivity was quantified in cells as a function of fluorescence intensity at **(A)** 1 h **(B)** 4 h **(C)** 24 h and **(D)** 48 h post-irradiation. A consistent reduction in TSPO expression was demonstrated across all time points after 0.01 Gy, relative to control, though was only statistically significant after 1 h (*p < 0.05). Contrastingly, an increase in TSPO expression was found in 2 Gy irradiated cells from 4 to 24 h, relative to control, though was not statistically significant (*p* > 0.05). **(E)** Representative images of irradiated and control microglia immunostained for TSPO (1 h post-irradiation). Data are represented as mean and standard error, and is the average of 3 experiments. Scale bar = 10 μm.

To focus on microglial TSPO expression after ionizing radiation exposure at different doses, primary microglia were harvested and cultured, then exposed to gamma radiation with a sham-irradiated control. Cells were fixed at different time points post-irradiation to observe the time-dependent effects on microglial reactivity. Fluorescence intensity quantification revealed a significant downregulation in TSPO expression after 0.01 Gy 1 h post-irradiation compared to controls (*p* < 0.05), whilst the other doses of 0.1 and 2 Gy remained relatively unchanged (*p* > 0.05, Figure 2). Subtle trends in this direction were also demonstrated at other time points after irradiation, including 48 h after irradiation, however, 1 h provided the most striking change in microglial TSPO expression. Consistent with the *in vivo* results, LDIR induces a downregulation of TSPO, and therefore microglial reactivity, after 0.01 Gy.

### Changes in Constitutive TSPO Expression in Cells of the Hippocampal Region After Irradiation

**FIGURE 3:**
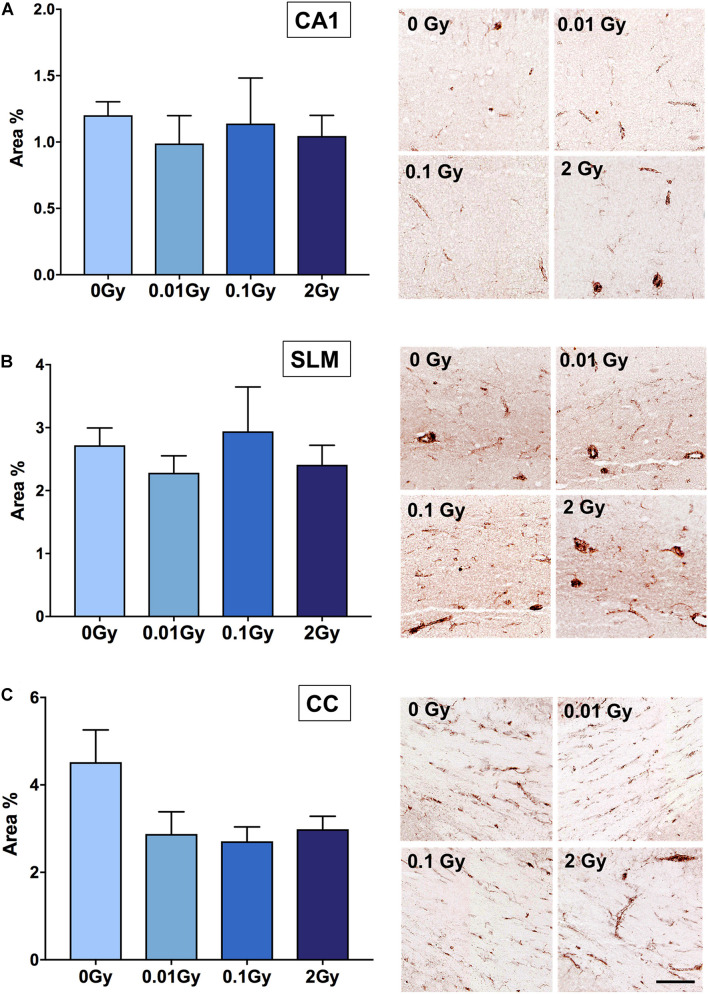
TSPO expression in hippocampal regions after gamma irradiation, measured as area% positive immunostaining with DAB. Regions measured were **(A)** CA1 (stratum radiatum), **(B)** stratum lacunosum moleculare (SLM), and **(C)** corpus callosum (CC). Whilst a trend toward a downregulation in TSPO expression was observed after 0.01 Gy compared to 0 Gy controls in the CA1 region, SLM and CC, this did not reach statistical significance (*p* > 0.05). A trend toward a downregulation after 2 Gy was also demonstrated in the CA1, SLM, and corpus callosum, though this also did not reach significance (*p* > 0.05). *n* = 4–5 animals/group. Scale bar = 40 μm.

**FIGURE 4:**
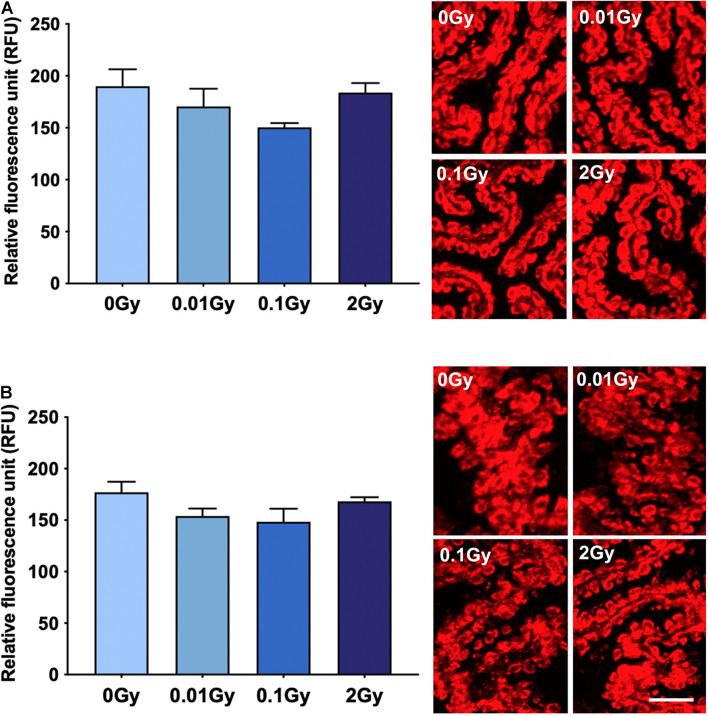
TSPO expression levels in regions of high constitutive expression after gamma irradiation, as measured by fluorescence intensity, including the **(A)** third ventricle ependyma, and **(B)** lateral ventricle ependyma. Gamma irradiation did not significantly alter TSPO expression levels in these regions/cells, though a trend toward a decrease after 0.01 and 0.1 Gy was observed (*p* > 0.05). *n* = 4–5 animals/group. Scale bar = 40 μm.

Having established an overall reduction in TSPO expression at the mRNA and protein level in the whole brain after 0.01 Gy, we investigated the cellular expression levels of TSPO using immunohistochemistry. The hippocampal region was chosen due to the radiation sensitivity of the various mitotically active cell populations in this region, including neural stem cells and vascular endothelial cells. The hippocampal regions with the highest area percentage TSPO expression included the stratum lacunosum moleculare (SLM) and the dentate gyrus (subgranular zone), as well as white matter regions adjacent to the hippocampus. Whilst these measures did not reach statistical significance for any radiation dose, a downward trend was observed after 0.01 and 2 Gy irradiation in the SLM, the corpus callosum, and the CA1 region (stratum radiatum), compared to controls (*p* > 0.05, Figure 3). In general, across the hippocampus, these radiation doses did not induce observable TSPO expression changes relative to controls in vascular endothelial cells of the region (*p* > 0.05, Supplementary Figure 2). To further examine TSPO expression in different cell types, fluorescence intensity measurements were performed in regions with known constitutively high TSPO expression, namely, the ependyma of the circumventricular system. In the third and lateral ventricular ependyma, TSPO expression trended toward a decrease at 0.01 and 0.1 Gy, compared to controls, whilst 2 Gy remained relatively unchanged (*p* > 0.05, Figure 4). Overall, LDIR induced some subtle changes in TSPO expression in vascular endothelial cells and ependymal cells, but appeared unchanged at the higher dose of 2 Gy.

### Differential Cytokine Levels After High and Low Dose Irradiation in *Tspo^+/+^* and *Tspo^–/–^* Plasma

To explore whether TSPO might play a role in systemic immune responses to radiation, we also irradiated *Tspo^–/–^* mice and compared them with irradiated *Tspo^+/+^* mice. Cytokine expression was measured in blood plasma 48 h post-irradiation. To capture pro-inflammatory effects and anti-inflammatory effects, IL-6 and IL-10 were measured using ELISA kits.

**FIGURE 5:**
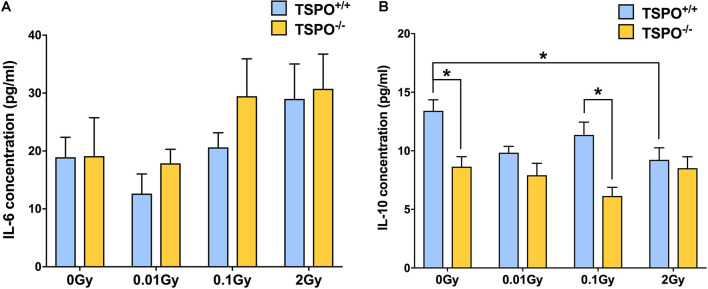
Effect of whole body gamma irradiation on plasma IL-6 and IL-10 expression levels in wild type (*Tspo^+/+^*) and TSPO knockout (*Tspo^–/–^*) mice. **(A)** ELISA analysis demonstrated greater IL-6 expression in both *Tspo*^+/+^ and *Tspo^–/–^* plasma after 2 Gy relative to 0 Gy controls, though this did not reach statistical significance (*p* > 0.05). A trend toward downregulation in IL-6 concentration after 0.01 Gy irradiation in *Tspo*^+/+^ plasma was observed, though levels in *Tspo^–/–^* plasma remained comparable to 0 Gy. **(B)** IL-10 concentration decreased in *Tspo^–/–^* plasma across all doses compared to *Tspo*^+/+^, though this was only statistically significant in control and 0.1 Gy plasma (*p < 0.05). IL-10 concentration was also significantly decreased in *Tspo*^+/+^ 2 Gy relative to 0 Gy control (*p < 0.05). Data are presented as mean and standard error, *n* = 6–9 mice/group.

At the baseline level of 0 Gy, no differences were seen between *Tspo^+/+^* and *Tspo^–/–^* animals (Figure 5A). After the lowest dose of 0.01 Gy, IL-6 concentration was downregulated in *Tspo^+/+^* animals, but did not reach significance (*p* > 0.05). Similar to levels of TSPO and Iba1 mRNA and protein, the lack of statistical significance in plasma IL-6 level after 0.01 Gy is due to low basal levels of inflammation in healthy animals, with little space for detection. There was no change in IL-6 levels observed in *Tspo^–/–^* animals. At the higher dose of 0.1 Gy irradiation, a non-significant increase in IL-6 expression appeared to be present in *Tspo^–/–^* mice, though the levels of IL-6 after 0.1 Gy in *Tspo^+/+^* mice were comparable to sham-irradiated control *Tspo^+/+^* animals (*p* > 0.05, Figure 5A). At the highest dose of 2 Gy, IL-6 was increased in both irradiated *Tspo^+/+^* and *Tspo^–/–^* mice compared to sham-irradiated (0 Gy) controls, though again this did not reach statistical significance (*p* > 0.05).

In contrast to IL-6, *Tspo^–/–^* animals at the baseline condition of 0 Gy had significantly lower IL-10 concentration (*p* < 0.05) than *Tspo^+/+^* animals (Figure 5B). At 0.01 Gy, IL-10 concentration decreased in *Tspo^+/+^* animals, reaching similar low levels as in *Tspo^–/–^* animals. At 0.1 Gy, IL-10 concentration in *Tspo^+/+^* animals increased slightly, while IL-10 concentrations in *Tspo^–/–^* animals decreased and were significantly lower than in *Tspo^+/+^* animals (*p* < 0.05) at this dose. In contrast to IL-6, the concentration of IL-10 in 2 Gy irradiated *Tspo^+/+^* plasma was found to be significantly decreased (*p* < 0.05), while the concentration in *Tspo^–/–^* animals remained stable. Taken together, we observed general increases in IL-6 above the dose of 0.01 Gy, while for IL-10, a significantly lower baseline concentration was found in *Tspo^–/–^* animals that appeared largely unresponsive to changing irradiation doses.

## Discussion

This study investigated the impact of both low and high dose gamma radiation on TSPO expression in brain tissue, primary microglial cultures, and systemic cytokines in *Tspo*^+/+^ and *Tspo***^–^***^/^***^–^** animals. We have demonstrated that acute high and low doses of gamma radiation differentially modulate TSPO expression, microglial activation, and the plasma cytokine concentrations of IL-6 and IL-10. LDIR (0.01 Gy) consistently induced a trend toward a downregulation in TSPO protein and mRNA expression both *in vivo* and *in vitro*, whilst the higher dose of 2 Gy remained similar to control.

Whilst this is the first study examining TSPO expression after ionizing radiation exposure, several studies have demonstrated microglial activation after high dose ionizing radiation exposure (Hwang et al., 2006; Han et al., 2016; Lumniczky et al., 2017), often leading to cognitive decline (Acharya et al., 2016; Krukowski et al., 2018; Allen et al., 2020). Pro-inflammatory cytokine expression and the activation of transcriptional mediators of inflammation, including AP-1 and NF-κB, have also been shown to increase after ionizing radiation exposure in the brain, accompanying microglial activation (Lee et al., 2010; Dong et al., 2015). It should be noted, however, that injuries, including those after radiation exposure, cause variable microglial responses with the emergence of different phenotypes, making common microglial activation markers, such as Iba1, difficult to interpret (Moravan et al., 2011). In this study, we used inducible TSPO expression as a proxy to measure microglial activation, while most of the constitutive TSPO baseline expression is confined to vascular endothelial cells (Betlazar et al., 2018; Notter et al., 2018b). We did not find evidence for an induced TSPO expression in activated microglia. Furthermore, Iba1 protein and mRNA expression provided no significant evidence of microglial activation at any dose.

In a previous study, in which whole body irradiated mice were exposed to a low dose of 0.063 Gy gamma radiation, the number of activated microglia decreased 24 months after exposure, whilst 0.5 Gy increased activated microglial Iba1, and induced a greater number of apoptotic and oxidative stress markers in the hippocampal region. A dose-dependent increase was also observed between 0.125 and 0.5 Gy in terms of protein carbonylation, a measure of oxidative stress, as well as increased Nrf2 expression at 0.5 Gy. Hence, the authors concluded that the reduced microglial activation after 0.063 Gy was suggestive of an anti-inflammatory effect of LDIR (Hladik et al., 2019). In this study, whilst we did not demonstrate any microglial changes *in vivo*, as well as the fact that the subtle TSPO expression changes in the parenchyma appeared to be from vascular sources, we did find alterations to systemic pro-inflammatory and anti-inflammatory cytokine expression levels, where 0.01 Gy reduced IL-6 expression, and the higher dose of 2 Gy induced decreased IL-10 expression. Interestingly, this was only observed in *Tspo*^+/+^ mice, demonstrating that TSPO might be necessary for the induction of the systemic anti-inflammatory effects of LDIR.

LDIR may decrease systemic IL-6 levels in mice, including in the brain, through its direct effects on the cells that release cytokines, and indirectly through a reduction of TSPO. It has been demonstrated that the level of TSPO expression positively correlates to the concentration of pro-inflammatory cytokines, including IL-6 (Karlstetter et al., 2014; Betlazar et al., 2016, 2020; Boyd et al., 2021). Reversely, increased pro-inflammatory cytokines may trigger an elevated level of TSPO expression. Cytokines in the brain can be synthesized locally by brain tissue and can also come from systemic blood cytokines. Microglia, invading inflammatory cells, vascular endothelial cells, pericytes, choroid plexus, astrocytes, and neurons in the brain can all produce cytokines (Galic et al., 2012). Systemic blood cytokines including IL-6 and IL-10, and except for IL-2, have been shown to cross the blood-brain barrier into cerebrospinal fluid and interstitial fluid spaces of the brain (Gutierrez et al., 1993; Banks et al., 1995). Efflux of cytokines from the brain to blood has also been observed (Banks et al., 1995). Several pathways for blood cytokines entering brain have been proposed, including passive transport of cytokines into the brain at sites lacking a blood-brain barrier, binding of cytokines to cerebral vascular endothelial cells to generate secondary messengers including prostaglandins and nitric oxide, carrier-mediated transport of cytokines into the brain through the blood-brain barrier, and afferent nerve terminals where cytokines are released (Watkins et al., 1995; Kronfol and Remick, 2000). The increased immune responses in mice induced by LDIR in this study may be due to decreased production of pro-inflammatory cytokines (IL-6) in the brain. TSPO levels in blood typically are higher than in brain, and thus may have a more pronounced effect on inflammatory status than in brain.

**FIGURE 6:**
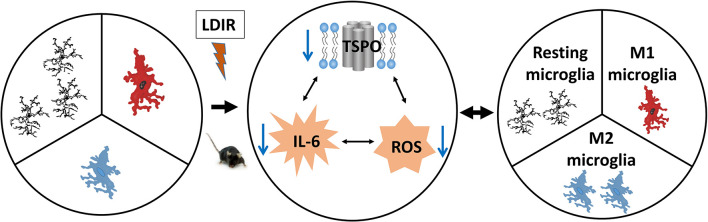
Low-dose ionizing radiation (LDIR) alters the microglial functional states by modifying levels of cytokines and ROS. LDIR decreases levels of TSPO, IL-6 and reactive oxygen species (ROS), which subsequently reduces the interaction among them, resulting in an increased ratio of polarized M2 microglia. The increased M2 microglia produce less pro-inflammatory cytokines (e.g., IL-6) and ROS while producing more anti-inflammatory cytokines (e.g., IL-10) and antioxidants.

As this study did not demonstrate changes in TSPO expression in microglia *in vivo* after irradiation, this suggests that the reduction in TSPO seen after LDIR was primarily from cells which constitutively express TSPO rather than from inducible expression. This is consistent with previous studies which have demonstrated a downregulation in TSPO expression from baseline in several neurological and psychiatric disorders, including PTSD (Gavish et al., 1996), depression (Chelli et al., 2008; Sarubin et al., 2016), and reduced TSPO ligand binding in PET imaging of recent onset schizophrenia (Notter et al., 2018a), despite the induction of systemic cytokine immune responses. We previously speculated that the reduced levels of TSPO that have sometimes been demonstrated in molecular imaging studies under certain subtle disease states reflect changes in vascular endothelial cells, which we found to have widely distributed, albeit low-level, expression of TSPO across the brain (Betlazar et al., 2018). In this study, we demonstrated subtle downregulation in the expression of TSPO in vascular endothelial cells of the hippocampus, particularly in highly vascularized regions, at doses up to 2 Gy. Endothelial cells are highly sensitive to radiation, and radiotherapy, amongst other factors, is mediated by microvascular sensitivity (Garcia-Barros et al., 2003). Radiation can induce several molecular pathways and processes in these cells, including the upregulation of cytokines, inflammatory cell recruitment, the involvement of NF-κB, and the production of reactive oxygen species (Venkatesulu et al., 2018). However, the responses of the brain’s vascular endothelial cells have been scarcely studied at doses under 2 Gy. Thus, given the link between the widespread constitutive TSPO expression in these cells, their ability to induce inflammatory responses and oxidative processes after irradiation, and the known involvement of TSPO in these processes, further studies are needed in order to gain insights into TSPO expression in this cell type after irradiation and its potential involvement in radiation-induced endothelial processes. It has been demonstrated that level of TSPO positively correlates with ROS production (Gatliff et al., 2014; Betlazar et al., 2016, 2020; Tu et al., 2016; Ilkan and Akar, 2018; Boyd et al., 2021). ROS and RNS can reversibly modulate TSPO expression (Loth et al., 2020). Decreased ROS levels in the brain induced by LDIR may be through its direct effects on brain cells and indirectly through a reduction of TSPO (Figure 6). Meng et al. (2020) demonstrated that decreased TSPO expression reduced oxidative stress, stabilized mitochondrial potential, inhibited the opening of the mitochondrial permeability transition pore, and prevented anoxia-induced apoptosis in rat cardiomyocytes (Meng et al., 2020). Pro-inflammatory cytokines and ROS can be positively inter-regulated (Yang et al., 2007; Rada et al., 2011; Agharazii et al., 2015). LDIR can directly decrease TSPO, pro-inflammatory cytokines and ROS, and depress their interaction, thus increasing immune responses (Figure 6).

A well-acknowledged link exists between mitochondrial metabolism, reactive oxygen species and microglial function, and this may modulate the heterogeneity of microglial phenotypes along the M1/M2 spectrum (Devanney et al., 2020). In line with this, TSPO has been used as a biomarker of microglial activation both *in vitro* and *in vivo*. Ligands binding to TSPO have been shown to modulate TSPO expression and shift the activation states of microglia between M1 and M2 pro- or anti-inflammatory states (Azrad et al., 2019; Monga et al., 2020). We have recently hypothesized that TSPO, through its regulatory role in mitochondrial energy metabolism and ROS generation, also exerts a generic immunomodulatory function (Ullah et al., 2020). At the molecular level, the link between radiation, mitochondrial processes, ROS, and inflammation needs to be further investigated by testing mitochondrial functions in microglia after irradiation. This is particularly interesting given the growing literature into mitochondrial responses in different cell types of the brain after irradiation, including the modulation of electron transport chain enzyme activity at 0.1 and 0.5 Gy (Kempf et al., 2015; Casciati et al., 2016), mitochondrial fission and fusion (Chien et al., 2015; Zhou et al., 2017), as well as the induction of oxidative pathways and oxidative damage to brain tissue, including lipid peroxidation, and increased glutathione and catalase levels after LDIR (Sharma et al., 2019). Further investigations into mitochondrial parameters in microglia after irradiation may also provide insights into the downregulation in microglial TSPO *in vitro* (where cells are in a constant state of activation) after LDIR.

Healthy brains are theoretically expected to show some, albeit minimal, basal level of TSPO expression reflecting the presence of activated microglia engaged in the active renewal and clearance of aged/dead cells. In the healthy brain, constitutive TSPO is predominantly expressed by endothelial cells in the blood vessels, ependymal cells in the ventricles, and neural stem cells, whereas TSPO expression in microglia and astrocytes is generally not seen, as demonstrated in our previous study (Betlazar et al., 2018). Since the present study demonstrated a trend toward downregulation of TSPO, we hypothesize that LDIR decreased the existing vascular baseline expression of TSPO. However, the fact that Iba1, too, showed a trend reduction indicates that even in the healthy brain that microglia, the cells that express Iba1, respond to LDIR. We hypothesize that this observation provides the foundation to examine whether LDIR can reduce the severity of neuroinflammatory tissue responses in induced localized neuroinflammation or injury, such as facial nerve axotomy, or induced global inflammation by lipopolysaccharide (LPS), or those prominently seen in traumatic brain injury or postulated for Alzheimer’s Disease.

TSPO is a widely used neuroinflammatory biomarker for *in vivo* studies, including positron emission tomography (PET) imaging in animals and patients. Inducible TSPO levels are dramatically increased in microglia under conditions characterized by neuroinflammation, such as multiple sclerosis (Banati et al., 2000) and glioma (Banati et al., 2014). The high contrast and diversity of TSPO expression levels in the brain—very low/none in the healthy brain and high under inflammatory conditions (particularly microglia)—make it a suitable marker for *in vivo* and *in vitro* study, including the study of radiation impact on the nervous system. While functional studies are not in the scope of this report, mitochondrial potential and ATP production is currently under further experimental investigation.

## Conclusion

In conclusion, this study demonstrated that acute LDIR reduced TSPO mRNA and protein expression in the brain, corresponding to a reduction in constitutive TSPO expression in vascular endothelial cells, though with no evidence of TSPO expression in parenchymal microglia. The reduction in TSPO expression is correlated with a switch from a M1 to an M2 functional state (Figure 6). TSPO expression, its role in mitochondrial processes and its altered expression in irradiated vascular endothelial cells of the brain, particularly after LDIR, warrants further exploration in future studies.

## Data Availability Statement

The original contributions presented in the study are included in the article/Supplementary Material, further inquiries can be directed to the corresponding author/s.

## Ethics Statement

All animal procedures were approved by the University of Sydney Animal Ethics Committee and the ANSTO Animal Care and Ethics Committee. All procedures were in accordance with the Australian Code of Practice for the Care and Use of Animals for Scientific Purposes (8th edition, 2013), and comply with the ARRIVE guidelines.

## Author Contributions

CB, RB, and G-JL: conceptualization. CB and G-JL: formal analysis. CB, RM, NH, BS, ED, and G-JL: investigation. CB, RM, NH, BS, ED, JD, and G-JL: methodology. CB, RM, and G-JL: project administration. RM, ED, JD, and G-JL: resources. RB and G-JL: supervision. CB: writing—original draft. RM, RB, and G-JL: writing—review and editing. All authors contributed to the article and approved the submitted version.

## Conflict of Interest

The authors declare that the research was conducted in the absence of any commercial or financial relationships that could be construed as a potential conflict of interest.

## Publisher’s Note

All claims expressed in this article are solely those of the authors and do not necessarily represent those of their affiliated organizations, or those of the publisher, the editors and the reviewers. Any product that may be evaluated in this article, or claim that may be made by its manufacturer, is not guaranteed or endorsed by the publisher.
