# Supplementary material for: Mitochondrial Translocator Protein (TSPO) Expression in the Brain After Whole Body Gamma Irradiation
Source: Front Cell Dev Biol. 2021 Oct 25;9:715444. doi: 10.3389/fcell.2021.715444 (PMC8573390; doi:10.3389/fcell.2021.715444)
Supplement: Supplementary file 1 [file Data_Sheet_1.PDF]

Supplementary Material

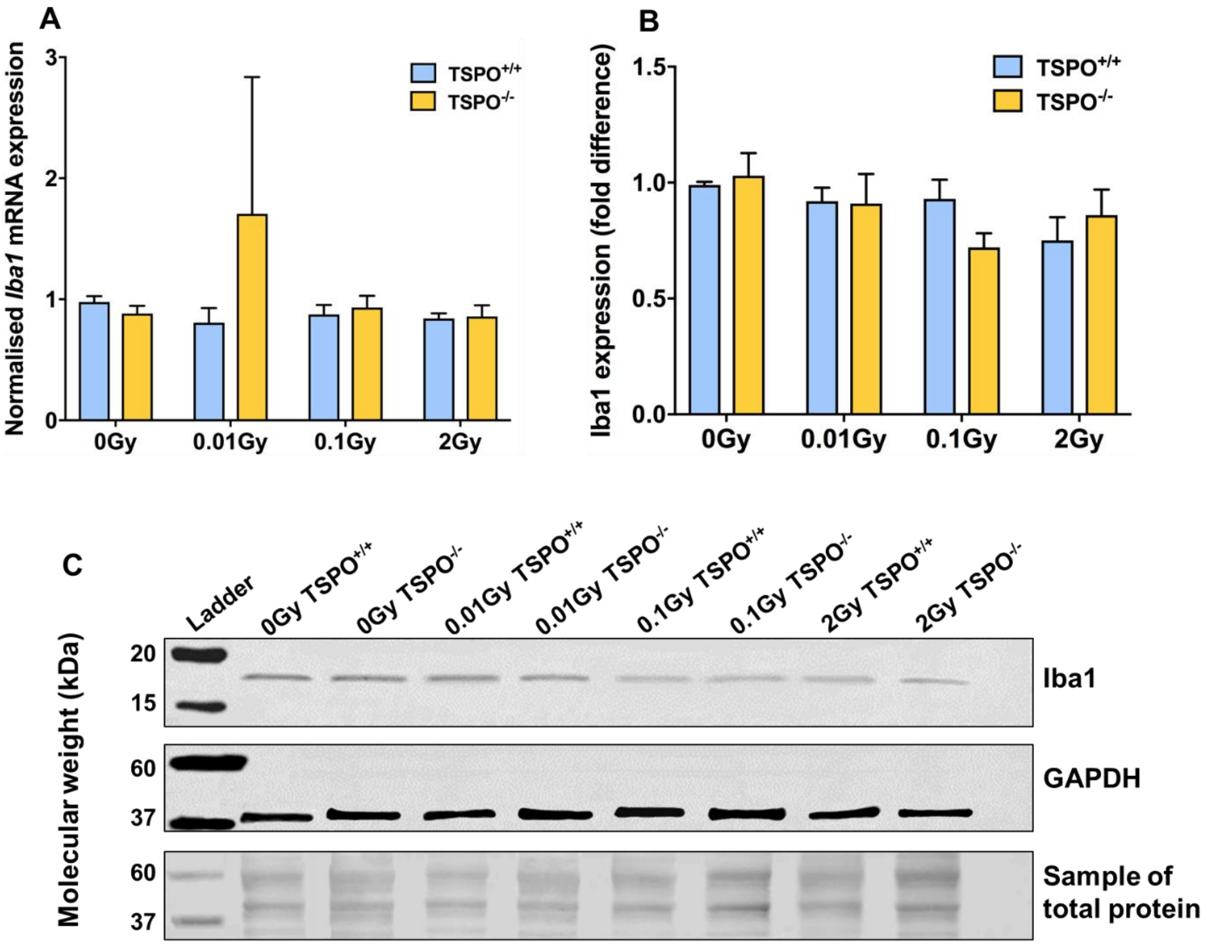

Supplementary Figure 1. Iba1 protein and mRNA expression in brain tissue from wild type (*Tspo*$^{+/+}$) and TSPO knockout (*Tspo*$^{-/-}$) mice following whole body gamma irradiation. **A)** *Iba1* mRNA expression (normalised to *Gapdh* and *Actin*) had a slight trend towards a decrease after 0.01 Gy in *Tspo*$^{+/+}$ tissue compared to control, though did not reach statistical significance (p>0.05). *Tspo*$^{-/-}$ tissue demonstrated similar *Iba1* mRNA expression levels to their *Tspo*$^{+/+}$ counterparts, except for at 0.01 Gy where 2 samples had demonstrable increases in Iba1 expression. **B, C)** Western blotting (normalised to total protein) confirmed the trend towards a downregulation in Iba1 protein expression after 0.01 Gy in

Tspo$^{+/+}$ tissue, as well as 0.1 Gy and 2 Gy, though did not reach statistical significance (p>0.05). No statistically significant difference was detected between *Tspo*$^{+/+}$and *Tspo*$^{-/-}$ tissue across doses (p>0.05). Blot images have been merged using bands from different membranes. Whilst the entire membrane was used for total protein normalisation, a section of the membrane is displayed in this image. Data are presented as mean and standard error, n=3-5/group.

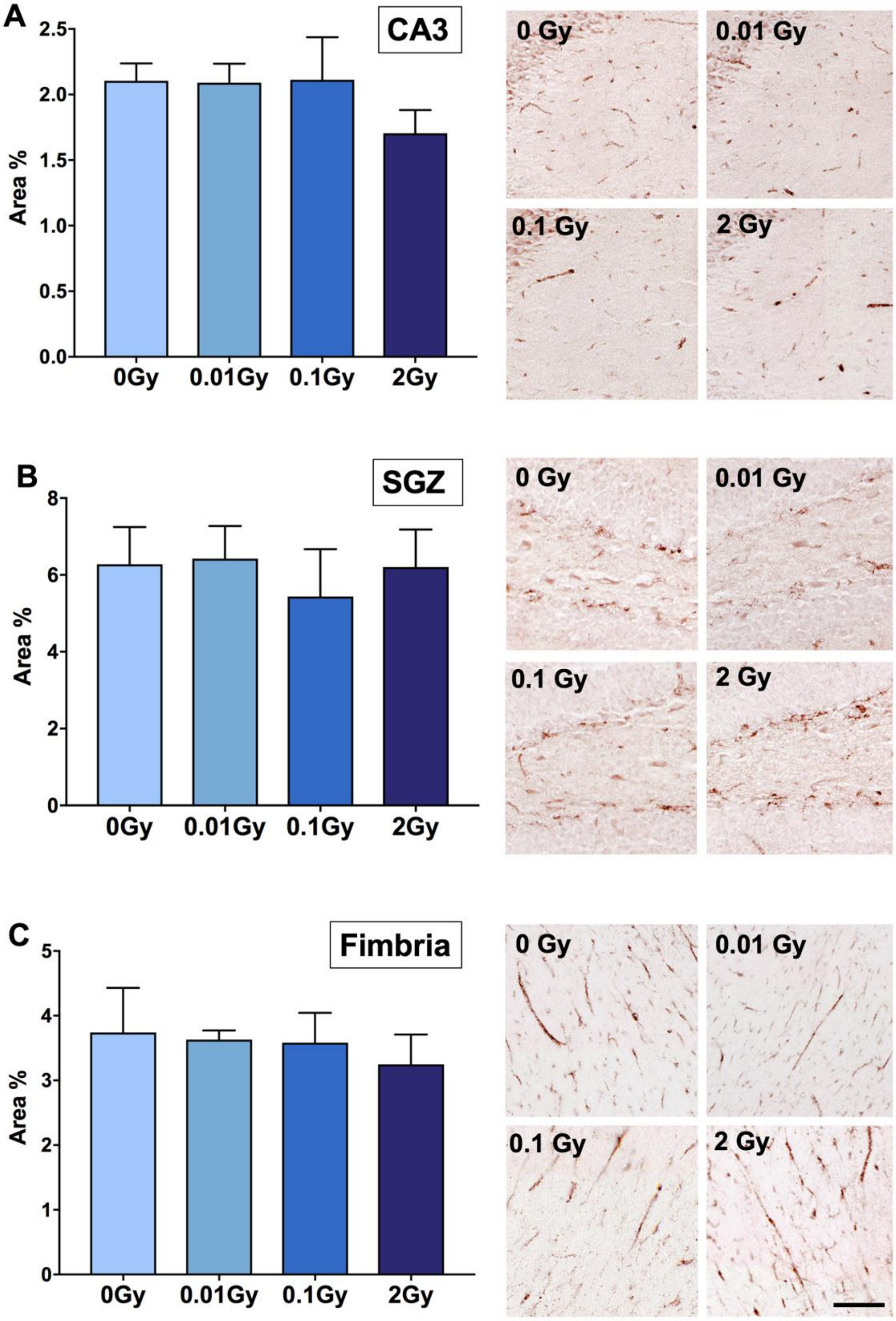

Supplementary Figure 2. TSPO expression in hippocampal regions after gamma irradiation, measured as area % positive immunostaining with DAB. Regions measured were **A)** CA3, **B)** subgranular zone (SGZ) of the dentate gyrus, and **C)** the white matter of the fimbria region. A trend towards a downregulation in TSPO expression was observed after 0.1 Gy compared to 0 Gy control

in the SGZ region, though this did not reach statistical significance ($p>0.05$). A trend towards a downregulation after 2 Gy was also demonstrated in the CA3 and fimbria, though this did not reach significance ($p>0.05$). n = 4-5 animals/group. Scale bar = 40 µm.

Supplementary table 1. RT-qPCR primers used in this study.

**Gene**	**Forward primer (5′ – 3′)**	**Reverse primer (5′ – 3′)**	**Reference**
Tspo	GGGAGCCTACTTTGTACGTGG	TGAAACCTCCCAGCTCTTTCC	
Iba1	ACAAAGAACACAAGAGGCCAACT	TGTGACATCCACCTCCAATCAG	
Actin	GGACCTGACGGACTACCTCATG	TCTTTGATGTCACGCACGATTT	1
Gapdh	CCATGGAGAAGGCTGGGG	CAAAGTTGTCATGGATGACC	2

Supplementary references

1. Morita, H. et al. Neonatal lethality of LGR5 null mice is associated with ankyloglossia and gastrointestinal distension. *Mol Cell Biol* 24 (2004): 9736-9743.
2. Dveksler, G. S., Basile, A. A. & Dieffenbach, C. W. Analysis of gene expression: use of oligonucleotide primers for glyceraldehyde-3-phosphate dehydrogenase. *PCR Methods Appl 1* (1992): 283-285.
